# Deciphering the Multi-Target Mechanisms of Sheshang Jiedu Decoction Against Snake Envenomation-Induced Acute Hepatic Dysfunction: An Integrated Multi-Omics Study

**DOI:** 10.3390/ph19071050

**Published:** 2026-07-07

**Authors:** Linfeng Wang, Jianqi Zhao, Fangwei Xia, Dianyun Sun, Qian Lei, Meilin Liu, Xiao Shi, Yang Yang, Chunhong Huang

**Affiliations:** 1 Department of Biochemistry and Molecular Biology, School of Basic Medical Sciences, Jiangxi Medical College, Nanchang University, Nanchang 330006, China; 2 Health Management Center, The Second Affiliated Hospital of Nanchang University, Xuefu Avenue 111, Honggutan District, Nanchang 330031, China; 3 Queen Mary School, Jiangxi Medical College, Nanchang University, Nanchang 330006, China

**Keywords:** animal toxins, *Deinagkistrodon acutus* venom, traditional Chinese medicine formula, acute liver injury, proteomics, network pharmacology

## Abstract

**Objective**: This study aimed to experimentally validate the hepatoprotective efficacy of Sheshang Jiedu Decoction (SSJDD) against *Deinagkistrodon acutus* (*D. acutus*) venom-induced acute liver injury (ALI) and systematically elucidate its multicomponent, multitarget mechanisms using an integrated multi−omics strategy. **Methods**: SSJDD constituents and serum-absorbed metabolites were profiled using UPLC-Q-Exactive HFX MS, and potential targets were predicted via network pharmacology. An in vivo model was established by intraperitoneally injecting Kunming mice with *D. acutus* venom, followed by a 7-day oral SSJDD intervention. The therapeutic efficacy was assessed by histopathological examination, serological analysis, and detection of oxidative stress markers in liver tissues. Label-free quantitative proteomics was performed on murine livers to map dynamic protein alterations and signaling cascades. **Results**: Integrated metabolomic and network analyses identified 15 primary active serum metabolites converging on core regulatory targets, including *TP53*, *AKT1*, and *CASP3*. In vivo, SSJDD dose-dependently ameliorated venom-induced lobular necrosis, suppressed elevated transaminases, and restored redox homeostasis without intrinsic hepatotoxicity. Quantitative proteomics revealed that venom triggered profound acute oxidative stress and coagulopathies, progressing to chronic metabolic disruption. SSJDD intervention substantially attenuated these proteomic alterations—reducing differentially expressed proteins by 84%—steering the hepatic microenvironment toward baseline homeostasis. Enrichment analyses demonstrated that these effects were primarily driven by modulating the coagulation-inflammation axis and the PI3K−Akt signaling pathway. **Conclusions**: SSJDD provides robust protection against *D. acutus* venom-induced ALI. Its active metabolites synergistically orchestrate hepatic repair and restore microenvironmental stability, primarily by targeting the PI3K−Akt pathway and regulating the coagulation-inflammation axis.

## 1. Introduction

Snakebite envenoming is a critical yet frequently neglected tropical disease (NTD), posing a significant public health challenge in rural communities across tropical and subtropical regions [1,2,3,4,5]. In China, this issue is particularly prominent in rural and mountainous areas, with annual reported snakebite cases ranging from 200,000 to 300,000, resulting in over 10,000 fatalities and hundreds of thousands of permanent disabilities [6,7,8,9]. Among medically significant venomous snakes in China, *Deinagkistrodon acutus* (*D. acutus*) is responsible for approximately 30% of severe snakebite cases in southern and central provinces, including Jiangxi, Hunan, and Fujian. Its venom, a complex mixture of snake venom metalloproteinases (SVMPs), phospholipases A_2_ (PLA_2_), and C-type lectins (Snaclecs), induces multi−organ damage, among which ALI is a prevalent and life−threatening complication [10,11]. Clinical data indicate that patients envenomated by *D. acutus* exhibit elevated serum alanine aminotransferase (ALT) and aspartate aminotransferase (AST) within 24 h of envenomation, and 20–30% progress to severe liver dysfunction or acute liver failure despite initial interventions [12]. While conventional first−line treatment relies on antivenom to effectively neutralize circulating free toxins, it possesses inherent limitations. Specifically, it cannot reverse established tissue damage such as hepatocyte necrosis, mitochondrial dysfunction, or inflammatory cascades triggered by venom−induced oxidative stress. Furthermore, potential hypersensitivity reactions and serum sickness, coupled with restricted availability in remote regions, limit its clinical utility [13,14]. Therefore, there is an urgent and compelling need to develop novel adjunctive drugs and therapeutic strategies that can mitigate the prolonged liver dysfunction, chronic inflammation, and impaired tissue repair caused by *D. acutus* venom, improve patient outcomes, and reduce the long−term burden of this severe injury.

Our previous proteomic studies demonstrated that the crude venom of *D. acutus* is dominated by SVMPs, 61.35% and snake Snaclecs, 28.47% as the core toxic components, supplemented by PLA_2_, 7.05% and trace amounts of coagulases and serine proteases [11]. From the perspective of traditional Chinese medicine (TCM), the venom of *D. acutus* is classified as a “toxic pathogen” with distinct characteristics of fire−toxicity, dampness, and blood stasis. According to TCM theory, this venom possesses fierce and rapid pathogenic properties, primarily manifesting as a “fire toxin” that consumes body fluids and stirs wind, leading to severe local swelling, blistering, and tissue necrosis [15,16]. Concurrently, the venom invades the blood system, causing “heat entering the blood chamber” and subsequent “blood stasis due to heat,” which corresponds to the hemorrhagic and coagulopathic manifestations observed clinically. The damp−toxic nature of the venom further complicates the clinical picture by impairing the normal functions of the spleen and stomach, leading to systemic disturbances. Given that the venom of *D. acutus* comprises multiple toxic components that simultaneously affect various tissues and organ systems, a single therapeutic agent targeting a specific molecular pathway is inherently limited in addressing the full spectrum of venom−induced pathology. The holistic and multi−target therapeutic approach characteristic of TCM, which emphasizes pattern differentiation and treatment based on overall syndrome analysis, offers distinct advantages in this regard. By employing composite formulas that clear heat, resolve toxins, cool blood, activate circulation, and dispel dampness, TCM can provide comprehensive and synergistic treatment that addresses both the local manifestations and systemic complications of *D. acutus* envenoming, thereby aligning with the current clinical need for integrated therapeutic strategies [17,18].

Sheshang Jiedu Decoction (SSJDD) was formulated by Xuchu Gong, a renowned traditional Chinese medicine practitioner at the Nantong Hospital of Traditional Chinese Medicine, based on decades of clinical experience in snakebite treatment. The formula comprises 14 medicinal herbs: *Lobelia chinensis* (Banbianlian), *Scleromitrion diffusum* (Baihuasheshecao), *Paris polyphylla* (Qiyeyizhihua), *Chrysanthemum morifolium* (Baijuhua), *Vincetoxicum pycnostelma* (Xuchangqing), Angelica dahurica (Baizhi), Imperata cylindrica (Baimaogen), *Periostracum Cicadae* (Chanyi), *Plantago depressa* (Cheqiancao), *Paeonia lactiflora* (Chishao), *Rheum palmatum* (Dahuang), *Salvia miltiorrhiza Bunge* (Danshen), *Rehmannia glutinosa* (Dihuang), and *Glycyrrhiza uralensis* (Gancao).

The formula follows TCM compatibility principles: *Lobelia chinensis*, *Scleromitrion diffusum*, and *Paris polyphylla* serve as the principal herbs to clear heat and detoxify [19,20,21,22]; *Chrysanthemum morifolium*, *Vincetoxicum pycnostelma*, *Angelica dahurica*, and *Periostracum Cicadae* clear heat and dispel wind to alleviate local swelling and systemic fever; *Salvia miltiorrhiza Bunge*, *Rehmannia glutinosa*, and *Paeonia lactiflora* cool blood and activate circulation to resolve blood stasis and improve liver microcirculation; *Imperata cylindrica*, *Plantago depressa*, and *Rheum palmatum* promote diuresis and relieve constipation to eliminate toxic metabolites; *Glycyrrhiza uralensis* harmonizes the actions of all herbs. Clinically, SSJDD has been shown to effectively alleviate systemic envenomation symptoms in the early and middle stages of snakebite, improve organ function, and prevent complications; however, its active components and underlying therapeutic mechanisms against *D. acutus*−induced ALI remain unclear, limiting its further clinical application and modernization [23,24,25,26].

In the present study, we employed an integrated approach combining network pharmacology and proteomic profiling to investigate the therapeutic basis and mechanism of action of SSJDD in the context of acute liver injury induced by *D. acutus* venom. Notably, this study is the first to identify the potentially active blood-activating and toxin-resolving components of SSJDD and to preliminarily characterize their multi-target effects. Furthermore, we experimentally validated the protective effects of SSJDD against venom-induced ALI and explored the possible mechanisms underlying this protection. This research provides a scientific foundation for understanding the therapeutic principles of SSJDD and offers insights for the development of evidence-based adjunctive therapies for *D. acutus* envenoming.

## 2. Results

### 2.1. Results of the Construction of Network Pharmacology

**Figure 1:**
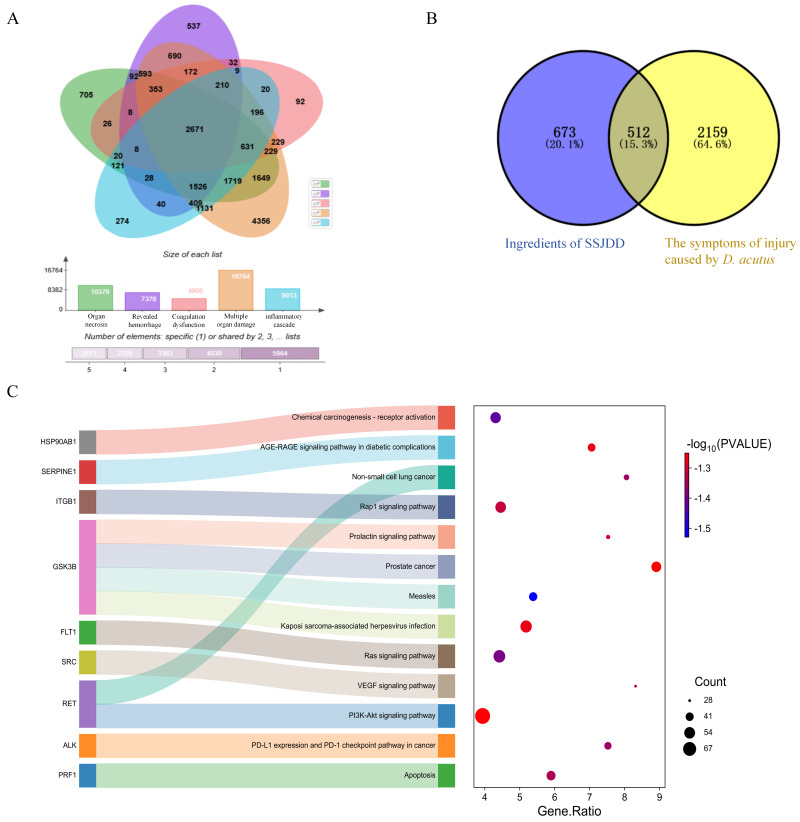
Network pharmacological analysis of SSJDD in treating venom of the *D. acutus*. (**A**) Disease intersection venn diagram; (**B**) SSJDD and venom intersection target diagram; (**C**) KEGG pathway enrichment.

To systematically explore the molecular mechanisms underlying *D. acutus* envenomation, potential targets across multiple primary pathological modules—including oxidative stress, inflammatory responses, and coagulation dysfunction—were integrated and analyzed (Figure 1A). Subsequent Venn diagram analysis revealed 2571 overlapping targets among these categories. This intersection highlights a complex pathophysiological network wherein oxidative and inflammatory cascades, vascular and hemostatic imbalances, and metabolic disruptions collectively drive acute liver injury. To further elucidate the therapeutic relevance of SSJDD against this venom−induced injury, its 1185 pharmacological targets were intersected with 2671 disease−associated targets (Figure 1B). The resulting identification of 512 shared targets—representing approximately 15.3% of the combined pool—demonstrates a robust molecular correspondence between the active components of SSJDD and the envenomation pathways. Taken together, these findings suggest that SSJDD mitigates *D. acutus* venom−induced pathology through extensive multi-target modulation.

To delineate the biological functions of these intersecting targets, GO and KEGG pathway enrichment analyses were subsequently performed. An evaluation of the top 10 enriched GO terms per category (Supplementary Figure S1A) revealed that the associated biological processes (BP) primarily involve chemokine and NF-κB signaling, T-cell receptor signaling, chemotaxis, cell cycle regulation, and cytokine responses. Concurrently, cellular component (CC) analysis predominantly highlighted the ABC−type transporter complex. In terms of molecular function (MF), the targets were significantly enriched for kinase, peptidase, and transcription factor binding, alongside ATPase, peptidase, and phosphatase activities. Furthermore, KEGG analysis (Figure 1C) mapped these targets to key pathways governing inflammation, apoptosis, and signal transduction; notable examples include the PI3K−Akt, VEGF, Rap1, Ras, AGE−RAGE, and PD−L1/PD−1 immune checkpoint pathways. Given their critical roles in modulating cytokine release, oxidative stress, and cell survival, the PI3K−Akt pathways are hypothesized to be particularly vital in attenuating snake venom-induced hepatocyte injury by maintaining metabolic homeostasis and cellular viability. Collectively, these enrichment analyses reinforce the premise that SSJDD mitigates venom−induced hepatic injury through integrated anti−inflammatory, antioxidative, and immunoregulatory mechanisms operating across multiple targets and pathways.

Finally, a PPI network was constructed to identify core molecular targets within the intersection network (Supplementary Figure S2A). Topological analysis identified *TP53* as the primary hub node; it exhibited the highest degree of connectivity, thereby underscoring its pivotal regulatory role. Additional prominent hub nodes—including *AKT1*, *CASP3*, *HSP90AA1*, *JUN*, *TNF*, and *IL6*—also displayed high connectivity, reflecting their broad involvement in diverse signaling cascades. Functionally, these targets act as key mediators of apoptosis, stress responses, inflammatory signaling, and cell survival. The robust interconnectivity among these proteins suggests that SSJDD likely modulates cell death and inflammatory pathways via a *TP53*-centered regulatory network, ultimately mitigating hepatic injury following *D. acutus* envenomation.

### 2.2. SSJDD Effectively Mitigates ALI Induced by D. Acutus Venom in Mice

**Figure 2:**
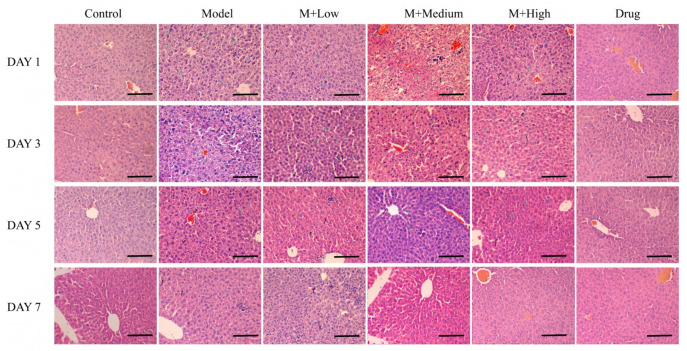
SSJDD effectively mitigates ALI induced by *D. acutus* venom in mice. Perform hematoxylin−eosin staining on liver tissue sections; Scale bar: 500 μm.

Histopathological evaluation (Figure 2) revealed that livers from the control group maintained a normal architecture across all time points, characterized by distinct lobular contours, orderly hepatocyte arrangement, and a lack of inflammatory infiltration or necrosis. In contrast, the model group exhibited progressive hepatic damage. Initial hepatocellular edema, focal degeneration, and inflammatory infiltration progressed to extensive necrosis, disruption of the hepatic cord architecture, and massive diffuse inflammatory cell infiltration. These changes were accompanied by marked congestion within the hepatic sinusoids and central veins. SSJDD treatment exerted dose−dependent hepatoprotection in venom−treated rats. Specifically, the low−dose group exhibited mild reductions in inflammatory infiltration; the medium−dose group displayed significantly decreased necrotic areas and partially restored lobular architecture; and the high-dose group demonstrated a largely orderly hepatocyte arrangement, minimal inflammation, and a near−normal overall hepatic structure. Furthermore, liver morphology in the SSJDD−only group was indistinguishable from that of the control group, presenting no pathological alterations. These findings suggest that SSJDD effectively alleviates *D. acutus* venom-induced acute liver injury in a dose-dependent manner, without exhibiting hepatotoxicity within the experimental dosage range.

### 2.3. SSJDD Protects the Liver from D. acutus−Induced Serious Oxidative Stress

**Figure 3:**
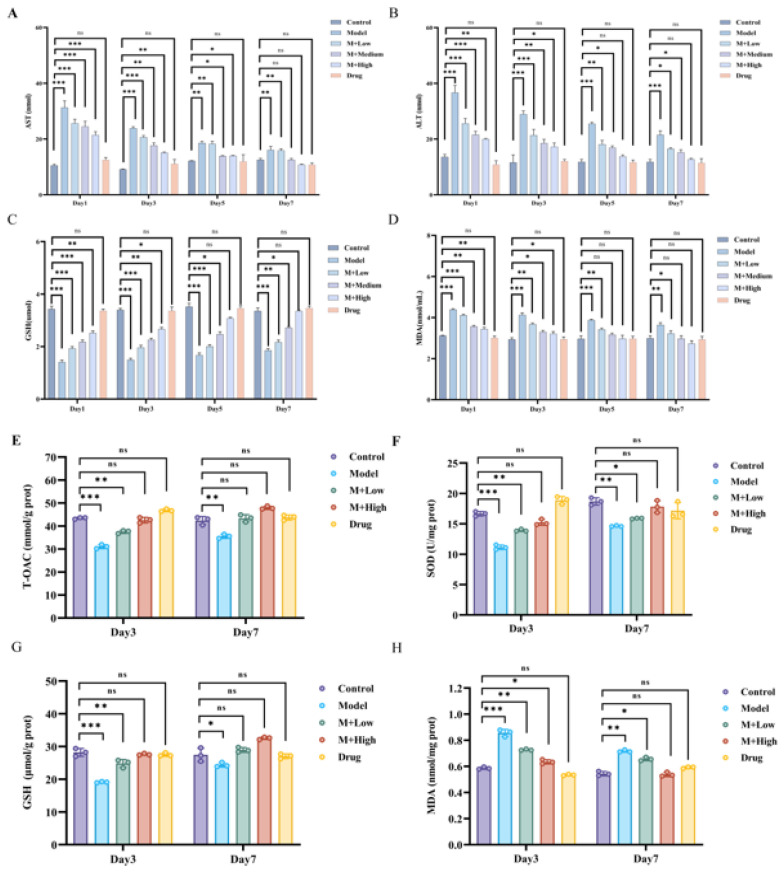
SSJDD treatment improves oxidative stress in the livers. (**A**–**D**) Serological liver function and oxidative stress indicators. (**E**–**H**) Liver tissue indicators of liver function and oxidative stress. (* *p* < 0.05, ** *p* < 0.01, *** *p* < 0.001, ^ns^ *p* > 0.05).

Serological analyses of key hepatic injury biomarkers (ALT and AST) and oxidative stress markers (GSH and MDA) (Figure 3A–D) corroborated the hepatoprotective effects of SSJDD. As shown in Figure 3A–D, the model group exhibited a significant and persistent elevation in serum ALT and AST levels over the 7-day observation period compared with the control group, indicating established acute liver injury. In parallel, liver tissue oxidative stress parameters (Figure 3E–H) revealed a marked increase in MDA content and substantial decreases in SOD activity, GSH and TOAC levels, confirming that venom−induced liver injury is accompanied by severe oxidative stress. Treatment with SSJDD at both low and medium doses, as well as the reference drug, dose-dependently reduced the venom-induced elevations in serum ALT and AST, an effect already discernible on Day 3 and more pronounced by Day 7. Concomitantly, SSJDD administration dose-dependently reversed the oxidative stress changes in liver tissue, lowering MDA levels and restoring SOD, GSH, and TOAC toward control values. The high-dose treatment demonstrated the most pronounced efficacy, effectively reversing venom-induced transaminase elevations and normalizing oxidative stress markers. Furthermore, the SSJDD−only group showed no significant serological differences from the control group at any time point, confirming the favorable safety profile of the intervention. Collectively, these results demonstrate that SSJDD exerts dose-dependent hepatoprotection by attenuating liver injury markers and restoring redox homeostasis, without manifesting evident toxicity.

### 2.4. UHPLC/MS Analysis of the SSJDD Herbal Extract and Serum Metabolites

#### 2.4.1. Chemical Structural Characterization of SSJDD

**Figure 4:**
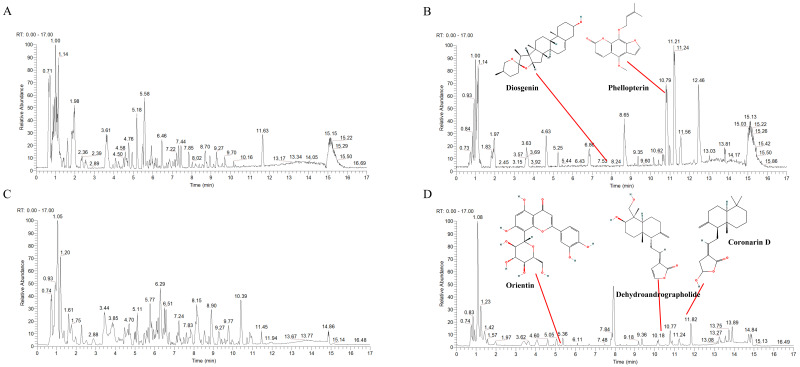
Chemical analysis of herbal extract and serum metabolites of SSJDD (**A**) Base peak chromatogram of SSJDD in positive ion mode; (**B**) Base peak chromatogram of SSJDD in negative ion mode; (**C**) Base peak chromatogram of SSJDD serum metabolites in positive ion mode; (**D**) Base peak chromatogram of SSJDD serum metabolites in negative ion mode.

Representative base peak chromatograms of the SSJDD extract and serum metabolites were acquired under both positive and negative ionization modes using UHPLC−Q Exactive HFX technology (Figure 4). Chemical constituents were cross-referenced against the PubChem database and relevant literature. Based on accurate molecular mass data obtained via high−resolution mass spectrometry (HRMS), putative chemical components were screened and preliminarily identified.

**Figure 5:**
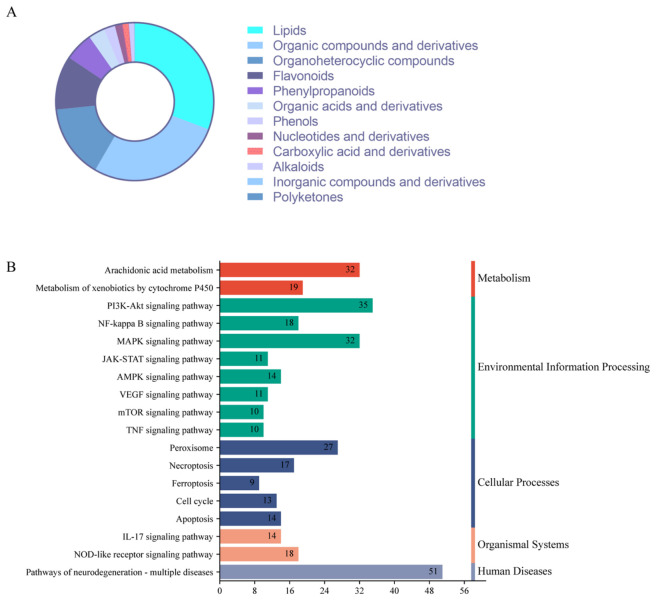
The network pharmacology analysis based on the serum metabolites of SSJDD. (**A**) Classification chart of SSJDD herbal extract; (**B**) KEGG enrichment analysis.

Chromatographic peaks and MS/MS fragmentation spectra of target compounds were extracted using Progenesis QI software, facilitating the inference of potential fragmentation pathways. HRMS-based metabolite profiling revealed that SSJDD comprises metabolites from 11 major categories (Figure 5A). The predominance of lipids and organic compounds indicates a multicomponent synergistic chemical profile. Specifically, lipids (30.72%) and organic compounds and their derivatives (27.64%) constituted the primary groups, followed by organic heterocycles (15.03%) and flavonoids (11.00%), which together form the structural core of the SSJDD metabolome. Phenylpropanoids (5.87%) and organic acids and their derivatives (3.37%) represented secondary components, whereas phenols, nucleotides, and alkaloids were present in lower proportions but exhibited considerable structural diversity.

By integrating the analysis of serum−absorbed metabolites under both ionization modes, 15 major active components were identified (Table 1), including key bioactive compounds such as cynaroside and dehydrovomifoliol. Given that previous studies have demonstrated the capacity of flavonoids and alkaloids to mitigate oxidative stress and inflammatory responses, these findings suggest that the complex chemical composition of SSJDD underpins its significant potential for alleviating snake venom-induced liver injury.

#### 2.4.2. Network Pharmacology Validation of Serum Metabolite Components in SSJDD

To elucidate the complex interactions of serum-absorbed metabolites, a PPI network was constructed and analyzed (Supplementary Figure S3A). The network nodes represent the absorbed metabolites, their putative target genes, and the associated signaling pathways. Topological analysis revealed that distinct metabolites are interconnected via multiple key targets and cascades, prominently featuring the MAPK, PI3K−Akt, JAK−STAT, and ErbB signaling pathways. These pathways are integral to cellular proliferation, apoptosis, stress response, and inflammatory regulation. Core targets—such as *TP53*, *AKT1*, *CASP3*, *JUN*, *TNF*, and *IL6*—exhibited high-degree connectivity and occupied central hub positions within the network. These central nodes likely mediate hepatocyte survival by modulating cascades associated with cellular injury and inflammation.

Preliminary network pharmacology analysis revealed that targets at the intersection of SSJDD and *D. acutus* envenomation-induced injury were primarily enriched in inflammation-regulating and cell survival-related pathways, including the PI3K−Akt and VEGF signaling pathways, among others. These findings suggest that SSJDD exerts its hepatoprotective effects via synergistic anti-inflammatory, antioxidant, and immunomodulatory mechanisms. Furthermore, in-depth KEGG enrichment analysis (Figure 5B) of the serum-absorbed metabolites demonstrated high congruence between these key pathways and the pharmacological activities of SSJDD’s bioactive constituents, including flavonoids, phenylpropanoids, and organic acids. Specifically, the active metabolites primarily modulate environmental information processing centered on the PI3K−Akt signaling axis (encompassing 35 enriched genes). They function in concert with the MAPK and NF-κB inflammatory cascades to suppress proinflammatory cytokine release and oxidative stress. Additionally, these compounds potentially facilitate venom detoxification by modulating arachidonic acid metabolism and cytochrome P450-mediated xenobiotic metabolism. Concurrently, they maintain hepatocellular redox homeostasis and viability by regulating pathways related to peroxisome function, apoptosis, and ferroptosis. In conclusion, the integrated network analysis demonstrates that SSJDD mitigates *D. acutus* venom−induced hepatic injury through a multicomponent, multitarget, and multipathway synergistic mechanism, with the PI3K−Akt signaling axis serving as the central therapeutic hub.

### 2.5. Results of Quantitative Proteomics Analysis

**Figure 6:**
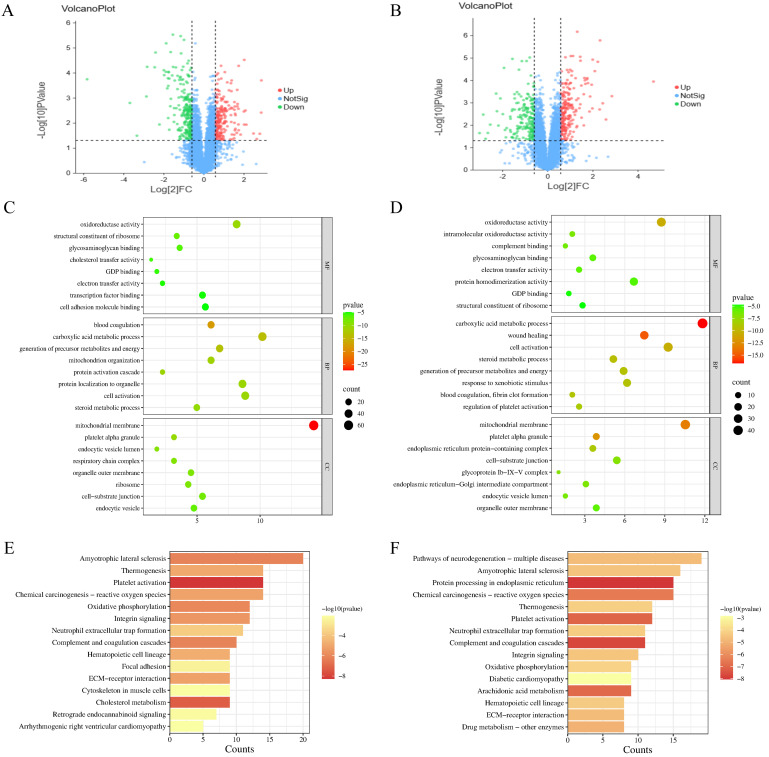
Comparative Quantitative Proteomic Analysis Between Model and Control Groups. (**A**,**B**) Volcano plot analysis of differentially expressed proteins; (**C**,**D**) GO enrichment analysis of quantitative proteomics data; (**E**,**F**) KEGG pathway analysis of quantitative proteomics data.

Serum hepatic injury biomarkers peaked on day 3 post−envenomation, indicating peak liver damage; thus, days 3 and 7 were selected for quantitative proteomic analysis. To investigate the temporal progression of hepatic injury induced by *D. acutus* venom, we compared the proteomic profiles of envenomated models with those of healthy controls. Differential expression analysis revealed profound and sustained alterations in the hepatic proteome (Figure 6A,B). On day 3, we identified 477 differentially expressed proteins (DEPs; 220 upregulated, 257 downregulated). This dysregulation persisted through day 7, with 422 DEPs (226 upregulated, 196 downregulated), indicating ongoing pathological changes driven by inflammation, cell death, and metabolic disruption.

GO and KEGG enrichment analyses (Figure 6C–F) further characterized the temporal evolution of the pathology. The acute injury phase on day 3 was marked by rapid-response mechanisms, oxidative stress, and mitochondrial dysfunction. Enriched cellular component GO terms included mitochondrial membrane integrity, respiratory chain complexes, and cell-substrate junctions. Key molecular functions involved ribosomal structural constituents, electron transfer activity, and cell adhesion molecule binding, whereas enriched biological processes featured blood coagulation, carboxylic acid metabolism, and protein activation cascades. KEGG analysis confirmed the significant enrichment of complement and coagulation cascades, oxidative phosphorylation, reactive oxygen species (ROS) signaling, and platelet activation pathways. By day 7, the pathological landscape had shifted toward a chronic stage characterized by tissue remodeling and metabolic reprogramming. This phase featured the enrichment of processes associated with mitochondrial organization, organellar protein localization, and steroid metabolism. Furthermore, KEGG analysis on day 7 revealed persistent dysregulation in pathways related to neurodegeneration, amyotrophic lateral sclerosis, thermosensitivity, retrograde endocannabinoid signaling, and cholesterol metabolism. Collectively, these findings reflect profound metabolic shifts, persistent cellular stress, and ongoing tissue repair mechanisms following the initial venom-induced insult.

**Figure 7:**
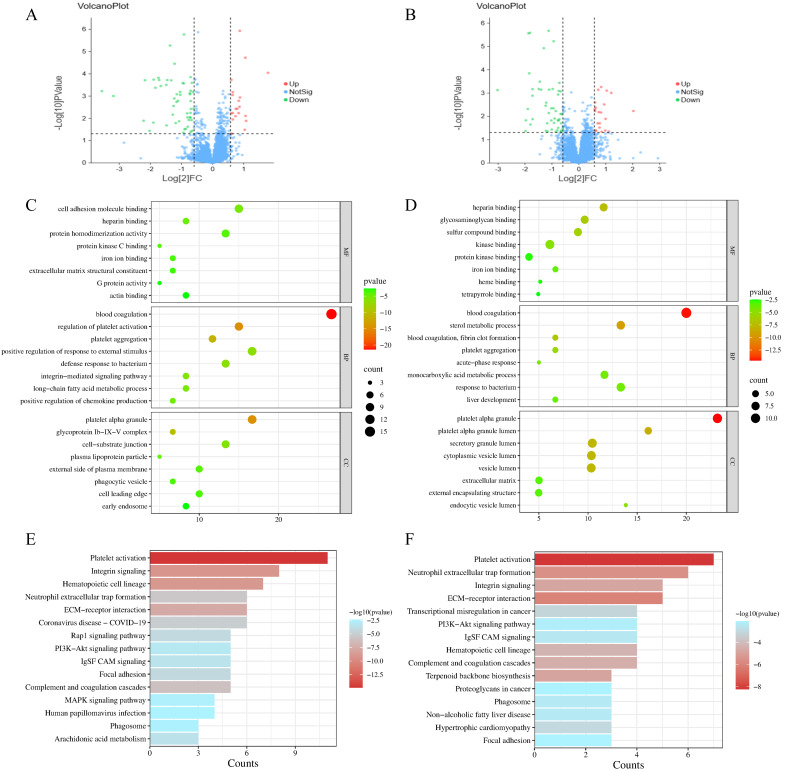
Comparative Quantitative Proteomics Between the Treatment and Model Groups. (**A**,**B**) Volcano plot analysis of differentially expressed proteins; (**C**,**D**) GO enrichment analysis of quantitative proteomics data; (**E**,**F**) KEGG pathway analysis of quantitative proteomics data.

In contrast to the widespread dysregulation in the envenomated model, SSJDD treatment profoundly reversed venom-induced injury and restored hepatic homeostasis. SSJDD administration substantially attenuated the scale of proteomic alterations, yielding only 74 DEPs (53 upregulated, 21 downregulated) on day 3 and 72 DEPs (51 upregulated, 21 downregulated) on day 7 (Figure 7A,B). This ~84% reduction in total DEPs compared to the model group, alongside a higher proportion of upregulated proteins, indicates a robust shift in the hepatic proteome toward baseline physiological conditions. This restoration was achieved primarily through the downregulation of pro−inflammatory and pro−coagulant factors, coupled with the upregulation of proteins vital for antioxidant defense, cellular repair, and metabolic homeostasis.

Enrichment analyses further elucidated the temporal therapeutic mechanisms of SSJDD (Figure 7C–F). On day 3, SSJDD primarily mitigated the acute pathological cascades triggered by envenomation. This was evidenced by the significant enrichment of biological processes related to blood coagulation, platelet activation, and extracellular matrix (ECM) organization. Notably, KEGG analysis revealed modulation of the complement and coagulation cascades—which were markedly activated in the model group—as well as regulation of the PI3K−Akt pathway, ECM-receptor interactions, and focal adhesion. These findings indicate that SSJDD counteracts coagulopathy and inflammation during the acute phase while actively promoting cellular survival and tissue structural integrity. By day 7, the therapeutic impact of SSJDD transitioned toward stabilizing the hepatic microenvironment and facilitating functional recovery. Modulation of the PI3K−Akt pathway persisted alongside the regulation of lysosome-related pathways, hematopoietic cell lineage differentiation, and proteoglycan binding. Additionally, the enrichment of processes related to cholesterol metabolism and cytoskeletal organization reflected the resolution of the metabolic reprogramming and structural disruptions that characterized the model group at this stage. This coordinated regulation of the coagulation−inflammation axis, coupled with restored metabolic and structural homeostasis, demonstrates that SSJDD acts through multiple synergistic mechanisms to reverse venom-induced pathological remodeling. Ultimately, these integrated actions restore hepatic function, mitigate long-term complications, and promote sustained recovery following *D. acutus* envenomation.

## 3. Discussion

Envenomation by *D. acutus* frequently precipitates ALI, a life-threatening complication. Although antivenom administration is the primary clinical intervention for neutralizing circulating toxins, it fails to ameliorate pre−existing tissue damage or suppress subsequent inflammatory cascades, highlighting a critical gap in current treatment protocols [27,28,29,30]. SSJDD, a TCM formula, has a long history of empirical use in snakebite management, primarily functioning to clear heat, detoxify, and promote blood circulation. This investigation offers the initial scientific corroboration of SSJDD’s hepatoprotective capacity against *D. acutus* venom. Through a multi-omics approach—combining network pharmacology, metabolomics, and quantitative proteomics with in vivo validation—we mapped the complex, multi-target mechanisms driving SSJDD’s therapeutic benefits [31,32,33].

Murine models in this study confirmed that *D. acutus* venom provokes swift and persistent hepatic impairment. This pathology manifested as extensive lobular necrosis, inflammatory cell infiltration, and significant surges in serum aminotransferases. Simultaneously, the venom precipitated severe oxidative stress, indicated by GSH depletion and MDA accumulation [34,35,36]. Subsequent oral intervention with SSJDD mitigated these venom-induced alterations dose−dependently. Notably, the high-dose regimen substantially rehabilitated hepatic architecture and restored redox homeostasis. Importantly, animals receiving only the botanical extract exhibited no histological or biochemical abnormalities, confirming the formula’s biosafety and absence of intrinsic hepatotoxicity at therapeutic dosages.

Our metabolomic analysis identified 15 serum−absorbed bioactive metabolites of SSJDD, primarily flavonoids (e.g., cynaroside) with well−documented anti-inflammatory and antioxidant properties [37,38,39,40]. Flavonoids are widely recognized for their robust anti-inflammatory and antioxidant activities. Topological network mapping indicated that these bioactives predominantly target core regulatory genes, including *TP53*, *AKT1*, *CASP3*, *TNF*, and *IL6*. Because these genes fundamentally govern apoptotic and inflammatory pathways, this molecular signature correlates strongly with the traditional TCM paradigm of “clearing heat and resolving toxins,” effectively bridging empirical herbal practices with contemporary molecular biology [41,42].

Quantitative proteomics uncovered a biphasic pathological progression of venom-induced liver injury: an early acute phase dominated by mitochondrial dysfunction, oxidative stress, and coagulation abnormalities, followed by a late chronic phase characterized by endoplasmic reticulum stress, metabolic reprogramming, and impaired tissue regeneration. Notably, this biphasic pattern mirrors the TCM pathogenesis of snake venom, which initially manifests as acute “fire toxicity” and progresses to chronic “blood stasis”. SSJDD intervention significantly reduced the number of differentially expressed proteins at both time points, restoring hepatic homeostasis [17,43,44,45,46].

Functional enrichment analyses identified the coagulation−inflammation axis and PI3K−Akt signaling cascade as the key pathways modulated by SSJDD. Venom exposure triggered excessive platelet activation and microvascular thrombosis, leading to ischemic hepatocyte necrosis. SSJDD effectively attenuated these early coagulopathies while potentially modulating the PI3K−Akt pathway to suppress apoptosis and pro-inflammatory cytokine secretion. This dual regulatory capacity highlights the advantage of TCM’s holistic approach over single-target therapies for complex venom-induced pathologies [47,48,49].

Certain limitations of the present study must be acknowledged. First, although primary serum-absorbed metabolites were characterized, the exact proportional contribution of individual constituents, such as cynaroside, to the aggregate hepatoprotective effect necessitates targeted validation in vitro and in vivo. Second, while multi−omics data strongly point to PI3K−Akt pathway involvement, subsequent investigations employing gene-knockout models or selective inhibitors are required to establish direct causality. Finally, translation of these preclinical findings to clinical practice will necessitate rigorously designed, multicenter randomized controlled trials to determine optimal dosing and long-term safety.

## 4. Materials and Methods

### 4.1. Snake Venom Collection and Preparation

The snake venom used in this study was obtained from *D. acutus*. Venom collection was conducted in strict accordance with ethical guidelines for the handling of wild animals, and all procedures were performed by trained personnel. Fresh venom was extracted by allowing it to pass through a cellulose-covered membrane into sterile 50 mL centrifuge tubes. Venom samples collected from individuals originating from the same geographical region were pooled, freeze-dried, and stored at −20 °C until further use.

### 4.2. Medications

A concentrated solution of SSJDD was developed by Shandong Jiechuang Health Industry Co., Ltd. (Jinan, China). It contains: *Lobelia chinensis* 30 g (batch No: C108250204, Hebei Anjia Pharmaceutical Co., Ltd., Baoding, China), *Scleromitrion diffusum* 30 g (batch No: 240901, Anhui Chengshun Pharmaceutical Co., Ltd., Tianchang, China), *Paris polyphylla* 15 g (batch No: 240522031, Bozhou Kuncheng Pharmaceutical Co., Ltd., Bozhou, China), *Chrysanthemum morifolium* 10 g (batch No: 240901, Anhui Chengshun Pharmaceutical Co., Ltd., Tianchang, China), *Vincetoxicum pycnostelma* 15 g (batch No: 8250420602, Hebei Baihe Health Pharmaceutical Co., Ltd., Baoding, China), *Angelica dahurica* 10 g (batch No: 20241111001, Shandong Dingshun Traditional Chinese Medicine Decoction Pieces Co., Ltd., Jinan, China), *Imperata cylindrica* 10 g (batch No: 2408033, Bozhou Jingcao Traditional Chinese Medicine Decoction Pieces Co., Ltd., Bozhou, China), Periostracum Cicadae 6 g (batch No: 065230802, Hebei Guozantang Pharmaceutical Co., Ltd., Cangzhou, China), *Plantago depressa* 30 g (batch No: 231001, Anhui Yiyuan Pharmaceutical Co., Ltd., Bozhou, China), *Paeonia lactiflora* 10 g (batch No: 240901, Anhui Chengshun Pharmaceutical Co., Ltd.), *Rheum palmatum* 10 g (batch No: C2532502001, Hebei Hehuachi Pharmaceutical Co., Ltd., Cangzhou, China), *Rehmannia glutinosa* 10 g (batch No: B2310057, Bozhou Jingcao Traditional Chinese Medicine Decoction Pieces Co., Ltd., Bozhou, China), *Glycyrrhiza uralensis* 5 g (batch No: 240501, Anhui Yiyuan Pharmaceutical Co., Ltd.), and *Salvia miltiorrhiza Bunge* 10 g (batch No: 240301, Anhui Yiyuan Pharmaceutical Co., Ltd.). All herbs were soaked, boiled, filtered, and concentrated to obtain low-, medium-, and high-concentration solutions of the SSJDD. The concentrations were 2.63 g/mL, 5.25 g/mL, and 10.5 g/mL respectively.

### 4.3. Animals Model and Ethics

Kunming mice (male, approximately 28–32 g, 6–8 weeks of age) were obtained from the Animal Center of Nanchang University (Nanchang, Jiangxi Province, China). According to procedures previously described, each experimental group comprised six mice, with a total of six groups in this study. (a) Normal saline (Control group); (b) Inject 0.2 LD_50_ *D. acutus* venom (Model group); (c) Venom + low-dose SSJDD group (M + Low group); (d) Venom + medium-dose SSJDD group (M + Medium group); (e) Venom + high-dose SSJDD group (M + High group); (f) The simple medium-dose SSJDD group (Drug group). Based on previous laboratory optimizations, mice in the envenomation groups received an intraperitoneal injection of *D. acutus* venom at a dose of 0.2 LD50. Six hours post-envenomation, the SSJDD treatment groups were administered their respective doses via oral gavage, a regimen that continued once daily for seven consecutive days. Concurrently, the Control and Venom model groups received an equivalent volume of distilled water via oral gavage. After administration through intraperitoneal injection, twenty-four hours later, blood samples were collected from the orbital area (500–600 μL for each mouse) by the ocular puncture method under isoflurane anesthesia. Blood was transferred to heparin-coated tubes, centrifuged at 3000 rpm for 10 min at 4 °C, and serum aliquots were stored at −80 °C. Subsequently, the cervical vertebrae were dislocated, and the mice were euthanized, and the main organs were collected. The serum and organs of the mice were collected for subsequent experiments. All animal experiments were conducted in accordance with the guidelines for animal experiments at Nanchang University and protocols approved by the Nanchang University Animal Ethics Committee (Ethics code: NCULAE−20220624042).

(Note: The equivalent dose [corresponding to the medium−dose group in mice, calculated based on the dose equivalence between humans and mice using a conversion factor of 9.1] = 9.1 × adult daily dose (3.86 g/kg·d) = 35.126 g/(kg·d). The SSJDD doses used in this study were clearly defined as follows: low dose = 17.563 g/(kg·d), medium dose = 35.126 g/(kg·d), high dose = 70.252 g/(kg·d). Administration concentration = equivalent dose [g/(kg·d)] × body weight (g) × multiplier ÷ gavage volume 0.2 mL.)

### 4.4. UPLC−Q−Exactive HFX MS Analysis of SSJDD

For the preparation and metabolomic analysis of the SSJDD samples, 0.5–1.0 mL of the sample was transferred into a centrifuge tube and mixed with an equal volume of extraction solvent (methanol/acetonitrile, 1:1). After vortexing for 60 s, the mixture underwent ultrasonic extraction at low temperature for 30 min, which was repeated twice to ensure complete extraction. The resulting solution was then incubated at −20 °C for 1 h to precipitate proteins, followed by centrifugation at 12,000 rpm for 20 min at 4 °C. The supernatant was collected, vacuum-dried, and reconstituted in 200 μL of 30% acetonitrile. After vortexing, the solution was centrifuged again at 14,000 rpm for 15 min at 4 °C, and a 2 μL aliquot of the supernatant was used for instrumental analysis.

Data acquisition was performed using an ultra-high-performance liquid chromatography coupled with a high−resolution quadrupole-Orbitrap mass spectrometry system (UPLC−Q Exactive HFX, Thermo Fisher, Waltham, MA, USA). Chromatographic separation was achieved on a Waters HSS T3 column (100 × 2.1 mm, 1.8 μm) with mobile phase A (0.1% formic acid in ultrapure water) and mobile phase B (0.1% formic acid in acetonitrile), at a flow rate of 0.3 mL/min, column temperature of 40 °C, and injection volume of 2 μL. The gradient elution program was as follows: 0–1 min, 100% A; 1–12 min, linear gradient to 5% A; 12–13 min, maintain 5% A; 13.1–17 min, re-equilibrate to 100% A. Samples were maintained at 4 °C in the autosampler throughout the analysis. Mass spectrometric detection employed an electrospray ionization (ESI) source with the following parameters: sheath gas 40 arb, auxiliary gas 10 arb, spray voltage +3.0 kV/−2.8 kV, ion source temperature 350 °C, and transfer tube temperature 320 °C. Data were acquired in both positive and negative ion modes using the Full MS−ddMS^2^ scan strategy, with a mass range of *m*/*z* 70–1050 Da, MS^1^ resolution of 70,000, and MS^2^ resolution of 17,500.

The raw data were processed using Progenesis QI software, which included baseline filtering, peak detection, sequence alignment, and retention time correction to generate a metabolite data matrix containing retention time, *m*/*z* values, and peak intensities. Finally, metabolites with available MS^2^ spectra were identified and annotated by matching them against HuiJun Biotechnology Co., Ltd.’s self−established herbal metabolite standard library, commercial databases, and characteristic fragmentation patterns [50,51,52].

### 4.5. Quantitative Proteomics Results Analysis

Liver tissue specimens were collected and sent to the Institute of Biomedical Innovation, Nanchang University (Nanchang, China), for label-free quantitative proteomic profiling. Protein extraction, enzymatic digestion, and LC-MS/MS analyses were conducted following established standard procedures. In brief, proteins were extracted, reduced with 10 mM dithiothreitol (DTT), alkylated using 55 mM iodoacetamide (IAA), and subsequently digested overnight with trypsin. The resulting peptides were purified via desalting and analyzed on a timsTOF Pro mass spectrometer coupled to a nanoElute UHPLC system. Mass spectrometric data were acquired in data−independent acquisition (DIA) mode. Raw spectra were processed in Spectronaut Pulsar 18.4 using the UniProt database with taxonomy restricted to *Mus musculus*. Protein identification was accepted when at least one unique peptide was detected, and quantification was performed at the MS/MS level. Differential protein expression analysis was based on peptide peak area measurements [11].

### 4.6. The Construction of Network Pharmacology

To construct a network pharmacology model, the formulation of SSJDD, containing 14 herbal components, was first analyzed. The chemical constituents of SSJDD were retrieved from the HERB database (http://herb.ac.cn/) and the Traditional Chinese Medicine Systems Pharmacology Database and Analysis Platform (TCMSP, https://tcmsp.91medicine.cn/). Compounds meeting the criteria of drug−likeness (DL) ≥ 0.18 and oral bioavailability (OB) ≥ 30% were selected as core active ingredients. Subsequently, the keywords “tissue necrosis,” “hemorrhage,” “coagulation dysfunction,” “multi-organ injury,” and “inflammatory cascade” were used to identify immune-related disease targets associated with *Deinagkistrodon acutus* (sharp-nosed pit viper) envenomation through the OMIM (https://www.omim.org/), GeneCards (https://www.genecards.org/), and DrugBank (https://go.drugbank.com/) databases. The screened protein targets were standardized using the UniProt database (https://www.uniprot.org/). Intersecting genes between the compound−related targets and the disease−related targets were analyzed using the STRING database (https://string-db.org/), and a protein–protein interaction (PPI) network was constructed. The resulting network was visualized and analyzed utilizing Cytoscape 3.10.3 software to identify the core targets based on topological parameter scoring. Finally, the curated core targets were imported into the Metascape platform (https://metascape.org/) for Gene Ontology (GO) and Kyoto Encyclopedia of Genes and Genomes (KEGG) enrichment analyses. Enrichment terms with a q-value < 0.05 were considered statistically significant, and results were ranked in descending order according to their *p*-values to identify the pathways most closely associated with the pharmacological action of SSJDD [53].

### 4.7. Serum Enzyme Analyses

Serum samples collected during the animal experiments were analyzed using commercial assay kits provided by Nanjing Jiancheng Bioengineering Institute (Nanjing, China). The biochemical parameters measured included alanine aminotransferase (ALT), aspartate aminotransferase (AST), malondialdehyde (MDA), and glutathione (GSH). All procedures were performed strictly in accordance with the manufacturer’s instructions to ensure the reliability and reproducibility of the results.

### 4.8. Liver Tissue Oxidative Stress Indicators

Liver tissue samples were homogenized in ice-cold physiological saline (1:9, *w*/*v*) and centrifuged at 2500 rpm for 10 min at 4 °C. The supernatants were collected for the determination of oxidative stress markers. Total antioxidant capacity (TOAC), superoxide dismutase (SOD), MDA and GSH levels in liver tissue homogenates were measured using specific assay kits (Nanjing Jiancheng Bioengineering Institute, Nanjing, China). All measurements were carried out following the manufacturer’s protocols to ensure reliability and reproducibility.

### 4.9. Histological Evaluation

Histological examination was performed with reference to established protocols, incorporating minor adjustments. Collected tissue specimens were immersed in 4% paraformaldehyde for a minimum of 24 h, sequentially dehydrated through graded ethanol, embedded in paraffin, and sectioned at a thickness of 5 µm. The sections were dewaxed with xylene and subsequently subjected to hematoxylin and eosin (H&E) staining for microscopic assessment [54,55].

### 4.10. Statistical Analysis

Data were expressed as mean ± standard deviation (SD). Statistical comparisons were conducted using one−way ANOVA followed by Tukey’s post hoc test when appropriate (SPSS version 23.0, IBM Corporation, Armonk, NY, USA). A *p*-value < 0.05 was considered statistically significant.

## 5. Conclusions

In conclusion, this research establishes that SSJDD substantially mitigates acute liver injury resulting from *D. acutus* envenomation. The decoction functions via an integrated network of bioactive metabolites that collectively neutralize oxidative stress, rectify coagulopathies, and dampen inflammatory responses. By primarily regulating the PI3K−Akt signaling pathway and the coagulation-inflammation axis, the formula facilitates hepatic repair and restores microenvironmental stability. These insights elucidate the contemporary pharmacological basis of a traditional botanical treatment, offering compelling preclinical evidence for the integration of SSJDD as a safe, adjunctive therapeutic option for severe snakebite management.

## Figures and Tables

**Table 1:** The active compounds of SSJDD.

Compound	Chemical Formula	Adduct	RT	*m*/*z*	Delta Mass (△m)	Structural Formula
Phellopterin	C_17_H_16_O_5_	[M+H, M+K, M+NH4, M+Na]	10.68	301.1060	−3.38	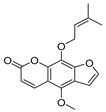
Cynaroside	C_21_H_20_O_11_	[M+H]	6.21	449.1068	−2.35	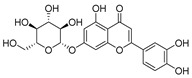
Alpha-Cembrenediol	C_20_H_34_O_2_	[M+NH4]	12.58	324.2886	−3.75	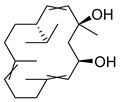
Ilexhainanoside D	C_36_H_56_O_11_	[M+H2O+Na]	7.43	705.3822	−0.49	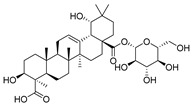
22-Acetoxyglycyrrhizin	C_44_H_64_O_18_	[M+H]	7.43	881.4135	−3.49	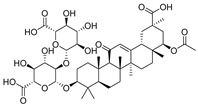
Diosgenin	C_27_H_42_O_3_	[M+CH3OH+Na+H2O]	7.67	487.3403	0.93	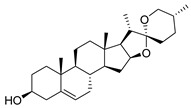
Glochidiolide	C_16_H_16_O_6_	[M+HCOO]	9.69	269.0800	−2.59	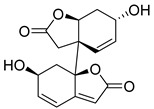
Dehydrovomifoliol	C_13_H_18_O_3_	[M−H]	8.22	267.1238	−0.14	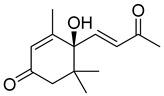
Ent-16beta,17-Dihydroxy-9(11)-Kauren-19-Oic Acid	C_20_H_30_O_4_	[M−H]	10.75	333.2072	0.11	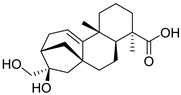
Jaceosidin	C_17_H_14_O_7_	[M−H, 2M−H]	7.95	329.0667	0.15	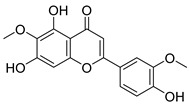
Emodin	C_15_H_10_O_5_	[M−H]	10.40	269.0455	−0.26	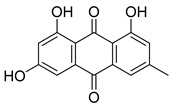
Rubiadin	C_15_H_10_O_4_	[M−H]	11.63	253.0507	0.37	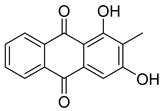
Coronarin D	C_20_H_30_O_3_	[M−H]	11.84	317.2121	−0.28	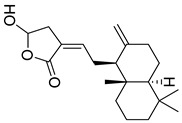
Dehydroandrographolide	C_20_H_28_O_4_	[M−H]	10.72	331.1895	−6.04	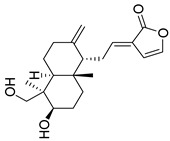
Orientin	C_21_H_20_O_11_	[M+HCOO]	5.32	447.0936	0.75	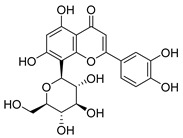

(Table 1. Adduct: Ionic adduct form; Retention time (RT); *m*/*z*: Measured mass−to−charge ratio; Delta Mass (△m): Relative standard deviation of quality; The structural formula was obtained from the PubChem website.)

## Data Availability

The original contributions presented in this study are included in the article/Supplementary Materials. Further inquiries can be directed to the corresponding authors.

## Supplementary Materials

The following supporting information can be downloaded at: https://www.mdpi.com/article/10.3390/ph19071050/s1, Figure S1A: Disease intersection venn diagram; Figure S2A: PPI network diagram; Figure S3A: PPI network diagram of “Component-Disease-Target”.
